# Impact of T-cell-specific Smad4 deficiency on the development of autoimmune diabetes in NOD mice

**DOI:** 10.1038/icb.2016.98

**Published:** 2016-11-15

**Authors:** Donghee Kim, Song Mi Lee, Hee-Sook Jun

**Affiliations:** 1 The Lee Gil Ya Cancer and Diabetes Institute, Gachon University, Incheon, Korea; 2 College of Pharmacy and Gachon Institute Pharmaceutical Science, Gachon University, Incheon, Korea; 3 Gil Medical Research Institute, Gil Hospital, Incheon, Korea

## Abstract

Type 1 diabetes results from autoimmune-mediated pancreatic beta-cell destruction and transforming growth factor-beta (TGF-β) is known to play a preventive role in type 1 diabetes in non-obese diabetic (NOD) mice. In this study, we investigated the role of Smad4, a key molecule for Smad-dependent TGF-β signaling, in T cells of NOD mice in the pathogenesis of autoimmune diabetes. We generated T-cell-specific Smad4 knockout (Smad4 tKO) NOD mice and assessed the pathological and immunological changes. Smad4 tKO showed earlier onset and increased incidence of diabetes than wild type (WT) NOD mice. Pathological features such as insulitis, anti-glutamic acid decarboxylase auto-antibody levels and serum IFN-γ levels were significantly increased in Smad4 tKO compared with WT NOD mice. Proportion and number of activated/memory CD4^+^ T cell were significantly increased in pancreatic lymph nodes of Smad4 tKO compared with WT NOD mice. However, the proportion and function of regulatory T cells was not different. Effector CD4^+^ T cells from Smad4 tKO were more resistant to suppression by regulatory T cells than effector cells from WT NOD mice. The proliferative potential of effector T cells from Smad4 tKO was significantly elevated compared with WT NOD mice, and activation of sterol regulatory element binding protein-1c (SREBP-1c) in T cells of Smad4 tKO NOD mice was correlated with this proliferative activity. We conclude that Smad4 deletion in T cells of NOD mice accelerated the development of autoimmune diabetes and increased the incidence of the disease by dysregulation of T cell activation at least in part via SREBP-1c activation.

Type 1 diabetes is a chronic disease, characterized by autoimmune-mediated destruction of pancreatic beta cells.^1^ It is known that T cells play a central role in the destruction of pancreatic beta cells.^2^ Both animal and human studies have demonstrated that the delicate balance of effector T (Teff) cells and regulatory T (Treg) cells determine the development of diabetes and insulitis.^1^ In the balanced state, pathogenic Teff cells sensitized by islet autoantigens can be expanded and activated in the target tissue and pancreatic lymph nodes (PLNs) and, in parallel, tolerization of naïve/Teff cells and expansion of Treg cells can occur. However, abnormalities of these Teff or Treg cells can lead to the development of autoimmune diabetes.^1^

TGF-β1 is a pleiotropic cytokine which belongs to the TGF-β super family and exerts multiple actions in various cell types.^3^ TGF-β is known to play an important role in differentiation, function and homeostasis of T cells.^4, 5^ In particular, TGF-β has immune suppressive functions and maintains peripheral tolerance.^6, 7, 8^ TGF-β KO mice in a mixed genetic background show severe inflammation and die within 3–4 weeks of age.^9^ Deficiency of TGF-β signaling in T cells results in the reduction of Treg cells^4, 10^ and the reduction of sensitivity in Treg cell-mediated suppressive responses.^11^ In animal models of type 1 diabetes, TGF-β suppresses the spontaneous onset of type 1 diabetes via expansion of Forkhead box (Fox)p3^+^ Treg cells within the islets of the pancreas.^12^ TGF-β also inhibits islet apoptosis and enhances proliferation and differentiation of Treg cells in non-obese diabetic (NOD) mice.^13^ In addition, serum TGF-β levels in type 1 diabetic patients is lower than in healthy controls,^14^ suggesting that TGF-β might play a preventive role in the development of diabetes.

TGF-β delivers signaling by binding to the TGF receptor II complex^15^ which phosphorylates the receptor-regulated Smads.^16^ The receptor-regulated Smad forms a complex by binding with Smad4, which subsequently translocates into the nucleus and regulates transcription of target genes.^17^ Therefore, Smad4 is a major pathway molecule for TGF-β signaling in T cells. However, when Smad4 is deleted in T cells of C57BL/6 genetic background mice, T-cell homeostasis is maintained without any observed symptoms.^18^ However, it is not known whether Smad4 plays a role in regulating the T cells of NOD mice, an animal model of autoimmune diabetes.

In this study we generated T-cell-specific Smad4-deficient mice in NOD genetic background and investigated the role of Smad4-mediated signals in T cell function required for the development of diabetes.

## Results

### Smad4 tKO NOD mice show earlier onset and increased incidence of type 1 diabetes

**Figure 1:**
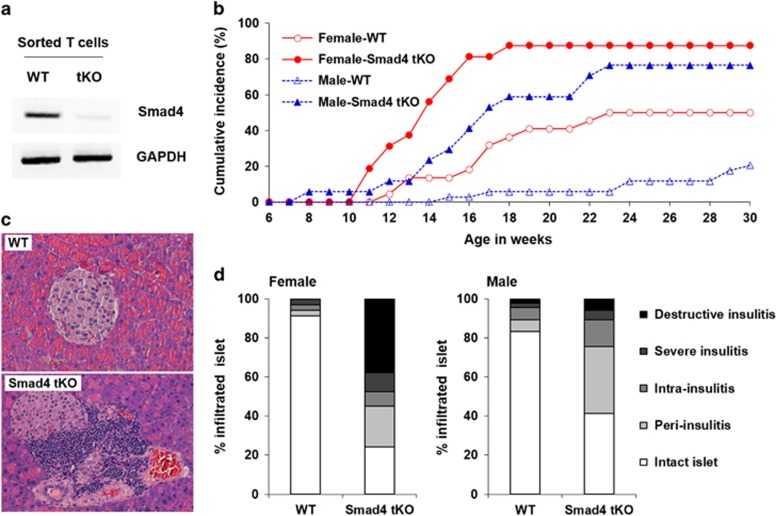
Smad4 tKO NOD mice show earlier onset and increased incidence of type 1 diabetes. (**a**) T cells were isolated from splenocytes in 12-week-old Smad4 tKO and WT NOD mice, and total RNA was extracted. The mRNA expression of Smad4 was analyzed by reverse transcription PCR. GAPDH mRNA expression was analyzed as an internal control. (**b**) Non-fasting blood glucose levels were measured in WT and Smad4 tKO NOD mice from 6 to 30 weeks of age. The onset of diabetes was defined as blood glucose levels over 250 mg dl^−1^ for two consecutive days. (Female WT *n*=22, Smad4 tKO *n*=16, male WT *n*=34, Smad4 tKO *n*=17, generation=9–11). (**c**, **d**) Pancreata were harvested from 8 to 9-week-old female and 15-week-old male WT and Smad4 tKO NOD mice and stained with hematoxylin and eosin. Insulitis score was categorized as follows; intact islet, peri-insulitis, intra-insulitis, severe insulitis and destructive insulitis. (**c**) Representative islet histology of male WT and Smad4 tKO NOD mice. (**d**) The data are the mean of the percentage of islets with each score. Number of male and female islets examined: WT=199, Smad4 tKO=189 and WT=208, Smad4 tKO=143, respectively. Number of male and female mice examined: WT=13, Smad4 tKO=16 and WT=4, Smad4 tKO=4, respectively.

We first confirmed the deletion of Smad4 in T cells by checking Smad4 messenger RNA (mRNA) expression by reverse transcription PCR analysis. Smad4 mRNA expression was not detected in sorted T cells from Smad4 T-cell knockout (tKO) NOD mice (Figure 1a). To investigate the effects of T-cell-specific Smad4 deletion on the development of type 1 diabetes, we assessed the cumulative incidence of diabetes by monitoring blood glucose levels in Smad4 tKO and wild-type (WT) NOD mice. We found that the cumulative incidence of diabetes by 30 weeks of age was 87.5% in female and 76.5% in male Smad4 tKO NOD mice, whereas it was 50% in female and 20.6% in male WT NOD mice (Figure 1b). In addition, Smad4 tKO NOD mice developed diabetes from 8 and 11 weeks of age in males and females respectively, whereas WT NOD mice developed diabetes from 15 and 12 weeks of age in males and females respectively (Figure 1b). When we examined islet infiltration of immune cells at 15 weeks of age in Smad4 tKO and WT NOD male mice, we found that islets from WT NOD male mice were mainly intact, while most of islets from Smad4 tKO showed insulitis (Figure 1c). We also found that the insulitis score of each mouse was higher in both male and female Smad4 tKO compared with WT NOD mice (Figure 1d). As male mice showed the most significant differences with respect to diabetes onset, we used male NOD mice for further studies.

### Smad4 tKO NOD mice show increased autoantibodies and inflammatory cytokines

**Figure 2:**
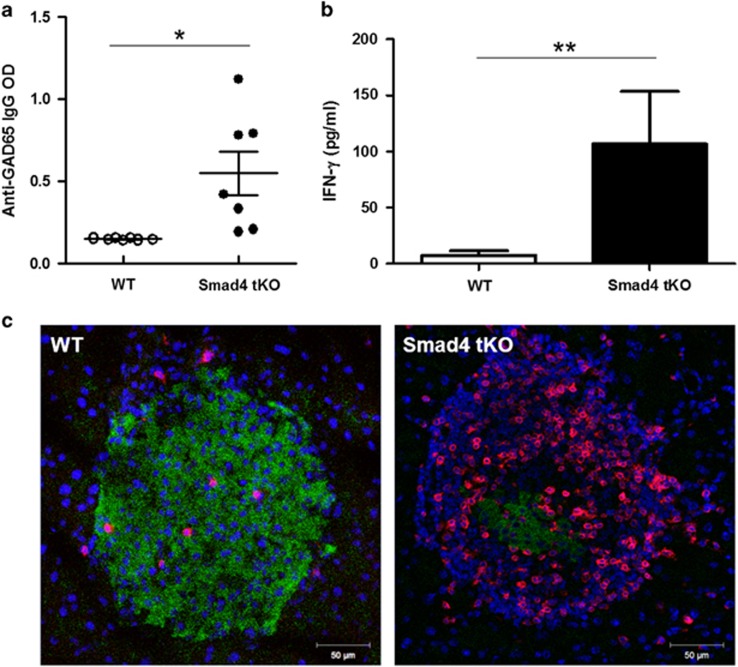
Smad4 tKO NOD mice show increased autoantibodies and inflammatory cytokines. (**a**) Sera were harvested from 8-week-old mice. Anti-GAD65 auto-antibody levels were measured using an enzyme-linked immunosorbent assay (Alpha Diagnostic Intl.) kit. Each circle represents an individual mouse (*n*=7/group). Bar graph shows the mean±s.d., *P<0.05. (**b**) Sera were isolated from 14 to 16-week-old mice. IFN-γ levels were measured using a mouse IFN-γ ELISA (Biolegend) kit. Values are means±s.d. (*n*=8 per group), *P<0.05. (**c**) Immunofluorescence staining for insulin (green) and CD3^+^ T (red) together with DNA staining with 4′-6-Diamidino-2-phenylindole (blue) in pancreatic cryosections from 15-week-old Smad4 tKO and WT NOD mice.

We next evaluated the level of anti-GAD65 autoantibodies in serum from 8-week-old Smad4 tKO and WT NOD mice. The level of anti-GAD65 autoantibodies in sera was significantly higher in Smad4 tKO NOD mice compared with WT NOD mice (Figure 2a). Serum IFN-γ levels were significantly higher (Figure 2b) and infiltration of T cells in the islets was also more severe (Figure 2c) in Smad4 tKO NOD mice than WT NOD mice. These results indicate that T-cell-specific Smad4 deficiency elicited pathological features for acceleration of the disease.

### The proportion of activated/memory T cells is increased, but the proportion of Treg cells is not changed in PLNs of Smad4 tKO NOD mice

**Figure 3:**
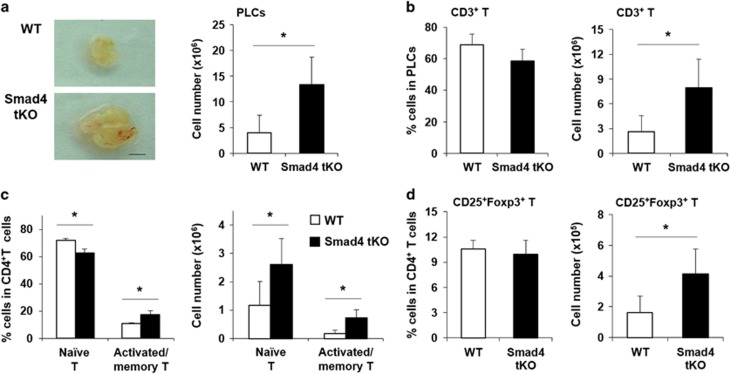
The proportion of activated/memory T cells is increased, but the proportion of Treg cells is not changed in PLNs of Smad4 tKO NOD mice. (**a**) PLNs were harvested from 12-week-old Smad4 tKO and WT NOD mice. Represented scale is 1 mm. PLCs were isolated from PLNs, and the cell numbers were measured. (**b**–**d)** PLCs were stained with anti-CD3, anti-CD4, anti-CD44, anti-CD62L, anti-CD25 and anti-Foxp3 antibodies and analyzed by FACS. (**b**) The proportion and absolute number of total T cells were determined as a percentage of CD3^+^ T cells in total PLCs. (**c**) Naïve cells were identified as CD4^+^CD44^low^CD62L^high^, and activated/memory T cells as CD4^+^CD44^high^ CD62L^low^ expression. The proportion and absolute number of naïve and activated/memory T cells were determined as a percentage of CD4^+^-gated T cells. (**d**) The proportion and absolute number of Treg cells were determined as a percentage of CD25^+^Foxp3^+^ cells in CD4^+^-gated T cells. Values are means±s.d. (*n*=5/group), *P<0.05.

As type 1 diabetes is considered to be a T-cell-mediated disease,^19^ we analyzed the T cell composition in PLNs from Smad4 tKO and WT NOD mice at 12 weeks of age. We found that the size and total cell number of PLNs were increased in Smad4 tKO compared with WT NOD mice (Figure 3a). Within the PLNs, the proportion of T cells was similar between Smad4 tKO and WT NOD mice (Figure 3b). However, the number of T cells was significantly increased in Smad4 tKO compared with WT NOD mice (Figure 3b). We then investigated the composition of T cell subsets in PLNs. The proportion of naïve CD4^+^ T cells (defined as CD44^low^ CD62L^high^) was significantly decreased in Smad4 tKO compared with WT NOD mice, whereas the proportion of activated/memory CD4^+^ T cells (defined as CD44^high^ CD62L^low^) were significantly increased in Smad4 tKO compared with WT NOD mice (Figure 3c). The number of both naïve CD4^+^ T cells and activated/memory CD4^+^ T cells was significantly increased in Smad4 tKO compared with WT NOD mice (Figure 3c). Interestingly, the proportion of Treg cells in CD4^+^-gated cells in Smad4 tKO was not different from WT NOD mice (Figure 3d), although the number of Treg cells was significantly increased in Smad4 tKO compared with WT NOD mice (Figure 3d). These results suggest that T cells of Smad4 tKO mice may have been more exposed to autoantigens compared with T cells from WT NOD mice.

### T cells in PLNs and pancreatic islets were activated in Smad4 tKO NOD mice

**Figure 4:**
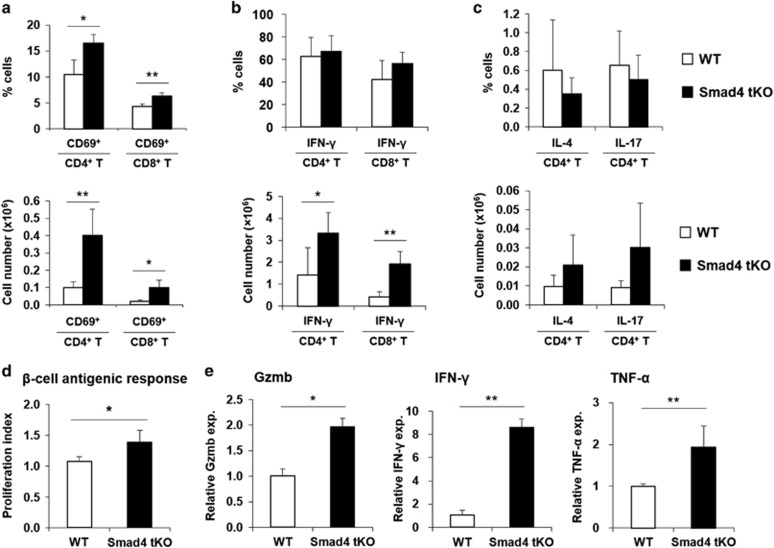
T-cell activation in PLNs and pancreatic islets was increased in Smad4 tKO NOD mice. (**a**–**d**) PLCs were isolated from PLNs of 12-week-old male Smad4 tKO and WT NOD mice. (**a**) PLCs were stained with anti-CD4, anti-CD8 and anti-CD69 antibodies and analyzed by FACS. The proportion and absolute number of CD69^+^ T cells were determined as a percentage of CD4^+^ T or CD8^+^ T cells. (**b**–**c**) PLCs were stimulated with phorbol 12-myristate 13-acetate (10 ng ml^−1^) and ionomycin (500 ng ml^−1^) for 12 h. Cells were stained with anti-CD4 or anti-CD8 antibodies and then intracellular stained with anti-IFN-γ, anti-IL-4 or anti-IL-17 antibodies. The proportion and number of (**b**) IFN-γ-expressing CD4^+^ or CD8^+^ T cells and (**c**) IL-4- or IL-17-expressing CD4^+^ T cells were analyzed by FACS. (**d**) PLCs were cultured with 50 μg ml^−1^ of NIT-1 lysate for 5 days and pulsed with [^3^H] thymidine. Proliferation index was calculated as counts per minute of NIT-1 lysate/counts per minute of medium control. (**e**) Pancreatic islets were isolated from 12 to 13-week-old male Smad4 tKO and WT NOD mice. The mRNA expression of granzyme B (Gzmb), IFN-γ and TNF-α was analyzed by quantitative PCR. Values are means±s.d., *n*=5 per group, *P<0.05, **P<0.01.

To assess T-cell activation in PLNs, we analyzed the proportion and absolute number of CD69-expressing CD4^+^ and CD8^+^ T cells. The proportion and number of CD69^+^CD4^+^ or CD69^+^CD8^+^ T cells were significantly increased in PLNs from Smad4 tKO compared with WT NOD mice (Figure 4a). As TGF-β potently inhibits Teff cell function and differentiation into Th1 and Th2 cells,^20^ we evaluated the cytokine production of PLN T cells by intracellular fluorescence-activated cell sorting (FACS) analysis. While the proportions of IFN-γ-expressing CD4^+^ or CD8^+^ T cells were similar between Smad4 tKO and WT NOD mice (Figure 4b), the number of IFN-γ-expressing cells was significantly increased in both CD4^+^ and CD8^+^ T cells from Smad4 tKO mice compared with WT NOD mice (Figure 4b). The proportion and number of IL-4- and IL-17-expressing CD4^+^ T cells were not different between Smad4 tKO and WT NOD mice (Figure 4c). To compare the antigen-specific T-cell response between Smad4 tKO and WT NOD mice, we tested the proliferation of pancreatic T cells using the cell lysate of the NIT-1 cell line, which is derived from an NOD mouse insulinoma^21^ as beta-cell antigen source. The proliferation index was significantly increased in pancreatic T cells from Smad4 tKO compared with WT NOD mice (Figure 4d). In addition, to investigate the effector function of T cells in pancreatic islets, we measured the mRNA expression of proinflammatory cytokines by quantitative PCR. The expression of granzyme B, IFN-γ and TNF-α was significantly increased in islets from Smad4 tKO compared with WT NOD mice (Figure 4e). These data collectively suggest that T cells in the PLN and infiltrated islets in Smad4 tKO NOD mice are in a more active state compared with WT NOD mice.

### Treg cells maintain suppressive activity, but Teff cells show resistance to regulation by Treg cells in Smad4 tKO NOD mice

**Figure 5:**
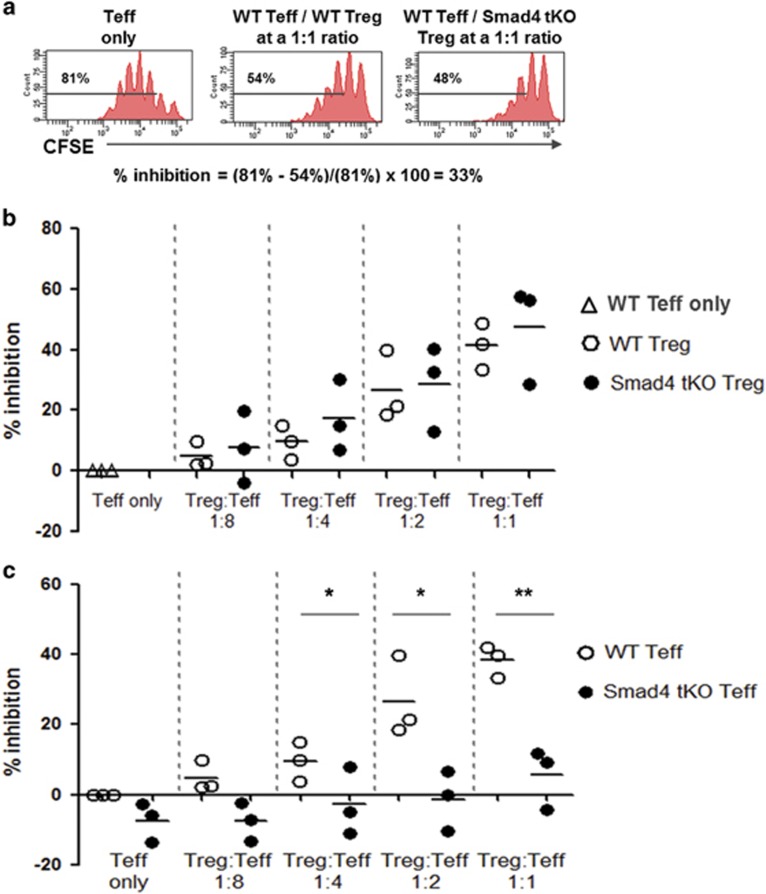
Teff cells show resistance to regulation by Treg cells in Smad4 tKO NOD mice. (**a**) Splenic and pancreatic Teff (CD4^+^CD25^−^ T) cells were isolated from 12-week-old WT NOD mice and were labeled with carboxyfluorescein succinimidyl ester (CFSE). For proliferation, Teff cells were stimulated with anti-CD3/28 coated beads for 72 h. Simultaneously, Treg (CD4^+^CD25^+^T) cells from WT or Smad4 tKO NOD mice were added at a ratio of 1:1 of Treg:Teff. Proliferation of Teff cells was assessed as CFSE dilution. Histograms show the percentage of proliferating Teff cells cultured alone or after gating out the Treg population. (**b**) Suppression assays using a Teff cells from WT cultured with Smad4 tKO or WT Treg cells. (**c**) Resistance assays using Treg cells from WT cultured with Smad4 tKO or WT Teff cells. The percentage of inhibition was determined by comparing the percentage of proliferating Teff cells cultured alone to the percentage of proliferating Teff cell in co-culture with Treg cells. Values are means±s.d. (*n*=3 per group), *P<0.05.

Because impaired Treg cells are linked to the development of autoimmune disease,^22^ we investigate the function of Treg cells from Smad4 tKO and WT NOD mice by examining their ability to inhibit the proliferation of Teff cells. Teff cells from WT NOD mice were incubated with Treg cells from WT or Smad4 tKO NOD mice during activation with anti-CD3/CD28 antibodies. There were no differences in inhibitory function of Treg cells between Smad4 tKO and WT NOD mice (Figures 5a and b), indicating that Treg cells of Smad4 tKO NOD mice were not functionally defective. We then examined whether Teff cells of Smad4 tKO NOD mice show any differences from WT NOD mice in proliferative responsiveness to Treg cells. Teff cells from Smad4 tKO NOD were significantly less inhibited than Teff cells from WT NOD mice (Figure 5c), indicating that Teff cells of Smad4 tKO NOD mice are less susceptible to suppression by Treg cells.

### Proliferation of Teff cells is increased and correlates with sterol regulatory element binding protein (SREBP)-1c activation in Smad4 tKO NOD mice

**Figure 6:**
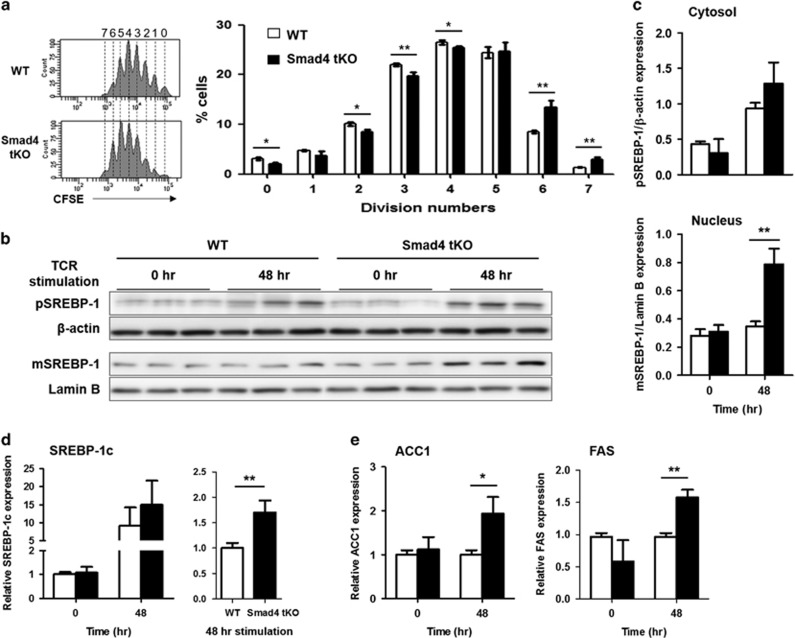
Proliferation of Teff cells is increased and correlates with SREBP-1c activation in Smad4 tKO NOD mice. Splenic and pancreatic Teff (CD4^+^CD25^−^ T) cells were isolated from 12-week-old Smad4 tKO and WT NOD mice. Teff cells were stained with carboxyfluorescein succinimidyl ester (CFSE) and were stimulated with anti-CD3/28 coated beads. (**a**) Proliferation of Teff cells was assessed as CFSE dilution at 72 h. The left panels show representative FACS profiles with numbers indicating number of cell divisions. (**b**, **c**) PLCs were harvested from Smad4 tKO and WT NOD mice and were stimulated with anti-CD3/28 bead activator for 48 h or unstimulated. The expression of mature (m) and precursor (p) SREBP-1 protein was analyzed by western blot. Mature/nuclear and precursor/cytosol SREBP-1 was normalized by lamin B and β–actin, respectively. Values were quantified by ImageJ software. (**d**, **e**) The mRNA expression of (**d**) SREBP-1c, (**e**) acetyl-CoA carboxylase 1 (ACC1) and fatty acid synthase (FAS) was analyzed by quantitative PCR after 48 h of stimulation. Values are means±s.d. (*n*=3), *P<0.05, **P<0.01.

TGF-β controls CD28-dependent cell growth and proliferation of CD4^+^ T cells through the Smad3 pathway.^23^ To investigate the effects of Smad4 deletion on T-cell proliferation, cellular division by T-cell receptor (TCR) stimulation was measured by FACS analysis. Teff cells from Smad4 tKO NOD mice divided more frequently compared with Teff cells from WT NOD mice, with the highest division rate at 72 h after TCR stimulation (Figure 6a). These results suggest that Smad4 plays a crucial role in inhibiting T-cell proliferation by TCR stimulation.

Lipogenesis is commonly achieved in proliferating cells by increased *de novo* fatty acid synthesis to support rapid cell membrane growth.^24^ SREBP, a major regulator of lipogenesis, is required for proliferation and reaction of Teff cells.^25^ Therefore, we examined the SREBP-1c mRNA and protein expression in pancreatic lymphocytes (PLCs) of Smad4 tKO and WT NOD mice after TCR stimulation. After 48 h of TCR stimulation, nuclear/mature SREBP-1c protein expression was significantly increased in Smad4 tKO compared with WT NOD mice (Figures 6b and c). After TCR stimulation, the mRNA expression of SREBP-1c was increased in PLCs from both Smad4 tKO and WT NOD mice (Figure 6d). In particular, we found that the mRNA expression of SREBP-1c in Smad4 tKO PLCs was significantly higher than WT PLCs after TCR stimulation (Figure 6d). In addition, the expression of acetyl-CoA carboxylase 1 and fatty acid synthase, which are target genes of SREBP-1c, was increased in Smad4 tKO compared with WT NOD mice (Figure 6e), indicating that the increased proliferation in Smad4 tKO T cells was positively correlated with an increase in activated SREBP-1c expression.

### Inhibition of SREBP-1 activation decreases T cell proliferation by TCR stimulation

**Figure 7:**
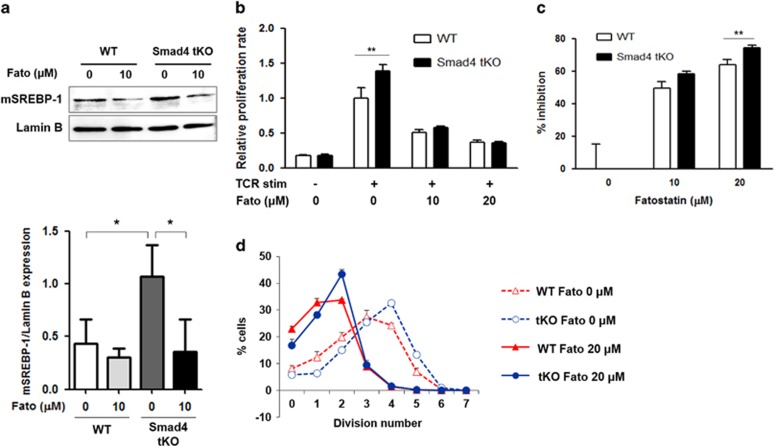
Inhibition of SREBP-1 activation decreases T cell proliferation by TCR stimulation. (**a**) PLCs were harvested from Smad4 tKO and WT NOD mice and were stimulated with anti-CD3/28 beads in the presence or absence of fatostatin (Fato, 10 μm) for 48 h. Cells were harvested and the expression of mature (m) SREBP-1 protein in the nuclear fraction was analyzed by western blot. Bar graph shows densitometry analysis of mSREBP-1 normalized with lamin B by ImageJ software. (**b**–**d**) PLCs were labeled with carboxyfluorescein succinimidyl ester (CFSE) and then stimulated with anti-CD3/28 beads in the presence or absence of Fato (10 or 20 μm) for 72 h. In the presence of Fato, (**b**) proliferation and (**c**) inhibition rate of T cells was determined by a cell counting kit-8 assay. (**d**) Cells were harvested and stained with anti-mouse CD4-APC-Cy antibody for T cell gating. Cells in the CD4^+^ T-cell-gated region were analyzed by FACS for proliferation based on CFSE dilution. The graph represents the percentage of cells undergoing the indicated number of divisions. Values are means±s.d. (*n*=3), *P<0.05, **P<0.01.

As the expression of SREBP-1 after TCR stimulation was further increased in the PLCs from Smad4 tKO NOD mice, we measured the SREBP-1c protein expression after TCR stimulation in the presence of fatostatin (Fato), which is an inhibitor of SREBP-1 activation.^26^ Although mature SREBP-1 expression without Fato was increased in PLCs from Smad4 tKO compared with WT NOD mice, this increase was blocked by the addition of Fato (Figure 7a). Next, we measured T cell proliferation after TCR stimulation in the presence or absence of Fato. T cell proliferation was reduced by Fato treatment in a dose-dependent manner in both Smad4 tKO and WT PLCs (Figure 7b). However, the T cell proliferation of Smad4 tKO PCLs was inhibited more than WT NOD PCLs (Figure 7c). In particular, the proliferation of rapidly dividing T cells from Smad4 tKO NOD mice was effectively inhibited in the presence of Fato similar to the level of T cells from WT NOD mice (Figure 7d). These results suggest that active proliferation of Smad4-deficient T cells is mediated by the SREBP-1c pathway.

## Discussion

Deciphering the molecular mechanisms that regulate T cell activation is a prerequisite for prevention of type 1 diabetes. Previous studies have shown that TGF-β plays an important role in the development of autoimmune diabetes.^12, 13^ TGF-β plasmid injection delays the development of diabetes in NOD mice,^13^ and TGF-β induces Foxp3^+^ T cells, restores self-tolerance and facilitates regeneration of beta-cell function in NOD mice,^27^ suggesting that TGF-β plays a preventive role in the development of type 1 diabetes.

TGF-β exerts biological functions through Smad-dependent and Smad-independent signaling pathways.^28, 29^ For Smad-dependent signaling, binding of TGF-β to the heterodimeric receptor TGFβRI and TGFβRII induces signaling cascades. Intracellular signaling pathways involve Smad proteins, including receptor-associated Smads (Smad1, 2, 3, 5 and 8), common Smad (Co-Smad, Sma4) and inhibitory Smads (Smad6 and 7). However, the specific immunological contribution of signaling molecules in TGF-β signaling networks are not clearly known in NOD mice. In this study, we investigated the role of Smad4 in T cells of NOD mice on the development of autoimmune diabetes. We found that T-cell-specific Smad4 deficiency in NOD mice accelerated and increased the onset of diabetes. The exacerbation of the disease was evidenced by increased islet inflammation, higher serum anti-GAD65 auto-antibody levels and increased inflammatory cytokine production at 12 weeks of age in Smad4 tKO mice compared with WT NOD mice. These results suggest that Smad4-mediated TGF-β signaling is important for the prevention of the development of type 1 diabetes in NOD mice.

As the draining lymph nodes, PLNs, are an important lymphoid organ of diabetic pathogenesis,^30^ we measured the T cell proportion and function in PLNs. We found that the proportion and number of activated/memory T cells were increased in Smad4 tKO NOD mice. In addition, T cells infiltrating the pancreatic islets showed a higher proliferative response to beta-cell antigens and higher expression of granzyme B. These results suggest that T cells in Smad4 tKO NOD mice might be more active in response to autoantigens. IFN-γ-secreting Th1 CD4^+^ helper T-cells are the major mediators of type 1 diabetes,^31^ and the balance of Th1 and Th2 cytokines is very important for the development of type 1 diabetes.^32^ Therefore, we analyzed the expression of Th lineage cytokines in T cells. T cells from Smad4 tKO NOD mice produced excessive IFN-γ, a Th1 cytokine, compared with T cells from WT NOD mice. However, the production of IL-4, a Th2 cytokine, was not significantly different between Smad4 tKO NOD mice and WT NOD mice. These results indicate that Smad4 in T cells might play a vital role in regulating IFN-γ production and Th1 differentiation.

In Smad4 tKO mice on C57BL/6 background, which we obtained to backcross with NOD mice, T-cell proliferation was significantly increased compared with WT T cells.^33^ This result is similar with our results obtained from Smad4 tKO mice on NOD background. However, in another report about T-cell-specific Smad4 deletion using CD4-Cre expression on C57BL/6 background, Smad4-deficient CD4^+^ and CD8^+^ T cells showed significantly less proliferation compared with WT T cells.^34^ In addition, one study reported that T-cell-specific Smad4 deletion in C57BL/6 mice exhibits normal homeostasis of T cells, including CD4^+^ and CD8^+^ T cells as well as natural Treg cells, in the thymus and secondary lymphoid organs.^35^ Therefore, even on the same C57BL/6 background, the function of Smad4 in T cells is controversial. Although it is not clear what affects these differences, original background of knockout mouse generation or the deleted exon site on Smad4 allele may result in this discrepancy.

Autoimmune disease can result from a loss of regulation of autoreactive T cells.^36^ Mechanisms of impaired Treg cell-mediated regulation include: (1) inadequate number of Treg cells, (2) defects in the function of Treg cells and (3) resistance of Teff cells to suppression by Treg cells.^36^ A reduced number or dysfunction of Treg cells has been suggested to contribute to the dysregulation of pathogenic Teff cells in autoimmune disease.^37, 38^ In contrast, other studies reported that there is no defects in Treg cells in type 1 diabetes.^39, 40^ We found that there was no differences in Treg cell numbers or function between Smad4 tKO and WT NOD mice. As Smad-independent pathways, such as the transforming growth factor beta-activated kinase 1-mediated pathway, are also important for the development, homeostasis and function of natural Treg cells,^41^ we speculate that natural Treg development may not depend on the Smad4 pathway in this case. Instead, Teff cells from Smad4 tKO NOD mice showed reduced sensitivity to Treg cells. Similarly, it was reported that downregulation of TGFRII in Teff cells in female NOD mice caused increased resistance to suppression by Treg cells.^42^ In addition, T cells from T-cell-specific TGFβRII- or Smad2/3-deficient C57BL/6 mice were not suppressed by WT Treg cells.^4, 10^ These results indicate that Smad-dependent signaling is involved in this resistance phenotype, which might be due to dysregulation of proliferation and excessive activation of Teff cells. However, Smad-independent signaling pathways, such as the transforming growth factor beta-activated kinase-mediated pathway, cannot be excluded for the development, homeostasis and function of Treg cells, as suggested in previous reports.^4, 41^

SREBPs are involved in lipogenesis for cell proliferation.^24^ SREBPs are known to be essential for meeting the heightened lipid requirements of membrane synthesis during blastogenesis, and Teff cells require SREBP for immune response.^25^ In addition, SREBP-1 is a mediator of TGF-β1 signaling in glomerular mesangial cells, and TGF-β1 induces the molecular interaction between SREBP-1 and Smad2, Smad3 and Smad4.^43^ Our results showed that the mRNA expression of SREBP-1c and its target genes such as fatty acid synthase and acetyl-CoA carboxylase 1 was significantly increased in T cells of Smad4 tKO NOD mice during TCR activation. In addition, SREBP-1c activation was increased and correlated with the proliferative activity of T cells, and inhibition of SREBP-1c activation by Fato, an inhibitor of SREBP cleavage-activating protein-assisted transport of SREBP-1,^44^ resulted in the reduction of proliferation by TCR stimulation. Therefore, Smad4 deficiency might increase SREBP-1c activation and affect the proliferative responses. Further studies are required to find out which molecular pathways are involved.

In summary, our results demonstrate that TGF-β signaling disruption by Smad4 deletion in T cells of NOD mice accelerates the development of type 1 diabetes and increases the incidence of the disease. Mechanisms include insensitivity of Teff cells to Treg cells in addition to higher proliferative activity of Teff cells in Smad4 tKO NOD mice. SREBP-1c activation in T cells was correlated with the proliferative activity in Smad4 tKO NOD mice. Therefore, Smad4 deficiency in T cells may affect metabolic pathway and desensitize pathogenic Teff cells, contributing to the promotion of type 1 diabetes.

## Methods

### Mice

Smad4^flox/+CD4-Cre^/NOD mice were generated by backcrossing Smad4^flox/flox;CD4-Cre^ (Smad4 tKO) mice on C57BL/6 background,^33^ from M Mamura (Kyoungbook University, Taegu, Korea) to NOD/ShiLtj for 9–11 generations. Subsequently, Smad4^+/+CD4-Cre^ (WT) mice and Smad4 tKO NOD mice were generated by brother–sister inbreeding. All experiments used age-matched male mice except for monitoring development of diabetes. Mice were housed at the animal facility of Lee Gil Ya Cancer and Diabetes Institute (CACU, Gachon University, Inchon, Korea), in accordance with ‘Guidelines for Animal for Users'. Blood glucose levels were monitored by glucometers (OneTouch Ultra, LifeScan, Wayne, PA, USA) from 6 to 30 weeks of age. Diabetes was diagnosed when the level of blood glucose went above 250 mg dl^−1^ in two consecutive measurements.

### Histological analysis

Tissues were fixed in 10% neutral buffered formalin and embedded in paraffin. The tissue sections (at 4–6 μm thickness) were stained with hematoxylin and eosin and images were analyzed with a light microscope. Islet infiltration was graded on a score as follows: intact islet, peri-insulitis, intra-insulitis, severe insulitis and destructive insulitis. For immunohistochemical analysis, pancreatic tissue cryosections (5 μm) were stained with anti-CD3 (BD Pharmingen, San Diego, CA, USA) or anti-insulin (Santa Cruz Biotechnology, Dallas, TX, USA) antibodies and 4′-6-Diamidino-2-phenylindole (Life Technologies, Carlsbad, CA, USA). Images were captured using a laser scanning confocal microscope (Carl Zeiss, Munich, Germany).

### Enzyme-linked immunosorbent assay

Serum IFN-γ and anti-glutamic acid decarboxylase (GAD)65 autoantibody were detected by Mouse IFN-γ (Biolegend, San Diego CA, USA) and anti-GAD65 (Alpha Diagnostic International, San Antonio, TX, USA) enzyme-linked immunosorbent assay kit, respectively.

### Antibodies, flow cytometry and cell sorting

Mechanically isolated PLC suspensions were incubated with anti-mouse CD16/32 (2.4G2, Biolegend) to block the Fc receptor before cell surface staining. All flow cytometric antibodies, unless otherwise noted, were purchased from BD Pharmingen. To identify subpopulation of lymphocytes, cells were stained with PE-CD3 (145-2C11), FITC-CD4 (GK1.5; Biolegend), APC-Cy7-CD4 (GK1.5), PE-CD4 (GK1.5), Pacific blue-CD8a (53-6.7), APC-CD44 (IM7), FITC-CD62L (MEL-14), APC-CD25 (PC61.5, eBioscience, San Diego, CA, USA) and FITC-CD69 (H1.2F3). Intracellular staining was performed using eBioscience's FOXP3 staining kit according to the manufacturer's instructions. Cells were stained by PE-Cy7-Foxp3 (FJK-16 s, eBioscience), PE-IL-17A (TC11-18H10.1, Biolegend), PE-IL-4 (11B11, BD Pharmingen) and PE-Cy7-IFN-γ (XMG1.2, BD Pharmingen). Flow cytometry data was acquired using a BD LSRII running Cell Quest Prosoftware (BD Biosciences, San Jose, CA, USA). Absolute counts of lymphocyte subsets were calculated by determining the proportion of positive cells to the gated cell population of interest and then multiplying this ratio by the number of total lymph node cells (little modified previous report^45^).

Pancreatic and splenic T cells were enriched using a mouse T-cell enrichment column (R&D System Inc., Minneapolis, MN, USA) and stained with anti-CD4 and anti-CD25 antibodies. Treg cells (CD4^+^CD25^+^) and effector T (Teff; CD4^+^CD25^-^) cells were isolated by the FACS AriaII system (BD Bioscience).

### Cell stimulation

To measure the proportion of cells producing cytokines, PLCs were stimulated with 10 ng ml^−1^ phorbol 12-myristate 13-acetate (Sigma-Aldrich, St Louis, MO, USA) and 500 ng ml^−1^ ionomycin (Sigma-Aldrich) for 6 h and then 1 μg ml^−1^ brefeldin A (eBioscience) was added for 6 h.

### *In vitro* T cell functional assay

To measure the T-cell proliferation, FACS-sorted Teff (CD4^+^CD25^−^) cells were stained with 5 μm carboxyfluorescein succinimidyl ester (Invitrogen, Carlsbad, CA, USA) and were stimulated with Dynabeads Mouse T-Activator CD3/CD28 (Life Technologies, 2 μl per well) for 3 days. For Treg inhibition assay, FACS-sorted Teff cells were stimulated and co-incubated in the presence or absence of FACS-sorted Treg (CD4^+^CD25^+^) cells at different ratios from 1:1 to 1:8 of Treg:Teff. The carboxyfluorescein succinimidyl ester fluorescence intensity was measured by a FACS LSRII flow cytometer using Cell Quest Pro Software (BD Biosciences) to determine the proliferation of Teff cells. In some experiments, T-cell proliferation was measured using Cell Counting Kit-8 solution (Dojindo, Kumamoto, Japan) according to the manufacturer's protocol.

### T-cells proliferative response to beta-cell antigens

To assess the proliferation of pancreatic T cells to autoantigens, we used lysates from the NOD insulinoma T-antigen-transformed-1 (NIT-1) cell line as a source of NOD mouse beta-cell antigens.^21^ The NIT-1 cell line (CRL-2055; American Type Culture Collection) was maintained in Ham's F-12K medium with 2 mm glutamine (Invitrogen) and 10% heat-inactivated fetal bovine serum (Invitrogen). The cells were harvested and resuspended in PBS, and lysed by repeated freeze-thaw cycles. The protein concentration of the lysate was measured by Pierce BCA Protein Assay Kit (Thermo Fisher Scientific lnc., Rockford, IL, USA). The lysate was dispensed into aliquots in sterile PBS. PLCs were isolated from 12-week-old Smad4 tKO and WT NOD mice and 1 × 10^5^ PLCs were seeded in 96-well round bottom plate. PLCs were cultured with or without the NIT-1 lysate at a concentration of 50 μg ml^−1^ for 5 days, and pulsed with [^3^H] thymidine (1 μCi per well, Amersham Biosciences, Pittsburgh, PA, USA) for 16 h. Then cells were harvested with a Micro Cell Harvester (Filter Mate Harvester, Perkin Elmer, Boston, MA, USA), and incorporation of ^3^H-thymidine was measured on a Tri-card 3110TR counter (Perkin Elmer, Boston, MA, USA). Proliferation indexes were calculated as counts per minute of NIT-1 lysate/counts per minute of medium control.

### Western blot analysis

PLCs were prepared and cultured in complete RPMI-1640 medium and stimulated with Dynabeads Mouse T-activator CD3/28 (Life Technologies) for 48 h. Cells were lysed and nuclear and cytosol fractions were prepared. Protein was resolved by sodium dodecyl sulfate–polyacrylamide gel electrophoresis and western blotting was performed with antibodies against β-actin, lamin B (Santa Cruz Biotechnology) and SREBP-1 (Abcam, Cambridge, UK). Signals were detected by using a Fujifilm luminescent image analyzer LAS4000 with an ECL detection kit.

### Reverse transcription PCR and quantitative real-time PCR

Total RNA was isolated from the sorted T cells or pancreatic islets, cDNA was synthesized and mRNA was amplified using specific primers. The amplified product was analyzed by agarose gel electrophoresis. GAPDH mRNA analyzed as an internal control. Quantitative real-time PCR analysis was performed using specific primers by SYBR Master Mix (TaKaRa Biotech, Kusatsu, Japan) and the CFX384 real-time PCR system (BIO-RAD, Hercules, CA, USA). Relative copy number was calculated using the threshold crossing point (Ct) as calculated by the ΔΔCt calculations. The primers used are listed in Supplementary Table 1.

### Statistical analysis

The data are presented as the mean±s.d. and analyzed for statistical significance using the unpaired Student's *t* test. The value of statistical significance was set at *P*<0.05.
