# Supplementary material for: Impact of T-cell-specific Smad4 deficiency on the development of autoimmune diabetes in NOD mice
Source: Immunol Cell Biol. 2016 Nov 15;95(3):287–96. doi: 10.1038/icb.2016.98 (PMC5364321; doi:10.1038/icb.2016.98)
Supplement: Supplementary Table 1 [file icb201698x1.docx]

**Supplemental Table 1. List of primers used for RT-PCR and qPCR**

**A. Primers for RT-PCR confirmation of gene transcription**

| **Gene** | **Forward primer** | **Reverse primer** |
| --- | --- | --- |
| GAPDH | 5'-AGTGCCAGCCTCGTCCCGTA-3' | 5’-TGAGCCCTTCCACAATGCCA-3’ |
| Smad4 | 5’-GGCCAGTTCACAATGAGCTT-3' | 5'-CCATCCACAGTCACAACAGG-3' |

**B. Primers for real-time qPCR analyses**

| **Gene** | **Forward primer** | **Reverse primer** |
| --- | --- | --- |
| Cyclophilin B | 5’-TGGAGAGCACCAAGACAGACA-3' | 5’-TGCCGGAGTCGACAATGAT-3' |
| SREBP-1c | 5’-GGAGCCATGGATTGCACATT-3' | 5’-GGCCCGGGAAGTCACTGT-3' |
| ACC1 | 5’-ACGCTCAGGTCACCAAAAAGAAT-3' | 5’-GTAGGGTCCCGGCCACAT-3' |
| FAS  Granzyme B  IFN-γ  TNF-α | 5’-GCTGCGGAAACTTCAGGAAAT-3'  5’-GCTAAAGCTGAAGAGTAAGGCCA-3’  5’- CGGCACAGTCATTGAAAGCCTA-3’  5’- CCAACGGCATGGATCTCAAAGACA-3’ | 5’-AGAGACGTGTCACTCCTGGACTT-3'  5’-CTTCCCCAACCAGCCACATA-3’  5’- GTTGCTGATGGCCTGATTGTC-3’  5’- AGATAGCAAATCGGCTGACGGTGT-3’ |
